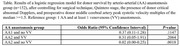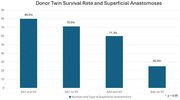# Oral Concurrent Session 8 – Fetus and Fetal Intervention: Saturday, February 1, 2025 8:00 AM – 10:15 AM

**DOI:** 10.1002/pmf2.12020

**Published:** 2025-01-05

## 86 | Innovative Transcardiac Access to the Fetal Cerebral and Pelvic Arteries for Diagnosis and Treatment

Michael A. Belfort^1^; Emma Van Den Eede^2^; Karen Chen^3^; Carolyn A Altman^3^; Timo Krings^4^; Peter T Kan^5^; Tomohiro Arai^2^; Wasinee Tianthong^2^; Walter Coudyzer^6^; Caitlin D. Sutton^3^; Samuel G G. McClugage, III^3^; William E Whitehead^3^; Magdalena Sanz Cortes^1^; Larry H Hollier^3^; Jan A. Deprest^2^; Luc De Catte^6^; Luc Joyeux^3^; Thierry Huisman^3^

*^1^Texas Children's Hospital and Baylor College of Medicine, Houston, TX; ^2^My FetUZ Fetal Research Center, Universitary Hospital Leuven, Leuven, Vlaams‐Brabant; ^3^Baylor College of Medicine and Texas Children's Hospital, Houston, TX; ^4^University Health Network, ON; ^5^UTMB, Houston, TX; ^6^Universitary Hospital Leuven, Leuven, Vlaams‐Brabant*

8:00 AM ‐ 8:15 AM

**Objective**: Direct femoral and carotid access is not possible in fetuses, limiting options for prenatal treatment of high flow life‐threatening anomalies (Vein of Galen Malformation, sacrococcygeal teratoma). Direct fetal cardiac puncture is used for intra‐uterine transfusion and fetal valvuloplasty. We hypothesized that direct cardiac catheterization would enable access to the fetal arterial system for diagnosis/treatment. Aim: develop a fetal sheep model to study feasibility and safety prior to human trials.

**Study Design**: 5 ewes at 120 days’ gestation (30‐week human equivalent) with 8 fetuses, underwent laparotomy with uterine exteriorization. Using ultrasound guidance (US) an 18G needle was placed through the uterine wall into the fetal heart via the apex of the left ventricle, and positioned under the aortic valve. A glidewire was advanced into the aortic arch. The needle was replaced with a 4F dilator. A 1.3F microcatheter was advanced over the glidewire into the aortic arch, and under fluoroscopy to (i) just proximal to the rete mirabile (vessel meshwork supplying the Circle of Willis) via the common carotid and internal maxillary arteries, or (ii) down the descending aorta to the femoral artery. N‐butylcyanoacrylate glue was then deposited occluding the target vessel. Sacrifice occurred 30 minutes after occlusion with CT and MRI assessment followed by autopsy (Fig1).

**Results**: All fetuses tolerated the procedure and survived 30 minutes. Intraoperative digital subtraction angiography and postmortem high‐resolution CT showed on‐target glue delivery in 7/8 (88%) cases. Postmortem MRI confirmed absence of focal brain and pelvis/limb ischemia/hemorrhage (Fig2).

**Conclusion**: This study demonstrates, in fetal lambs, feasibility/safety of a new procedure using direct cardiac catheterization (US guided) to access the arterial circulation in the brain and pelvis (fluoroscopy guided). This paves the way for other multiple‐method imaging guided procedures, including devascularization of high‐flow lesions, diagnostic blood sampling, biopsy, and drug and cell delivery and represents a new space for fetal research/therapy.

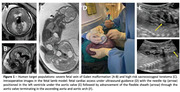

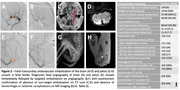

## 87 | Predicting Outcomes in Congenital Diaphragmatic Hernia with Discordant Fetal MRI and Ultrasound Prognostic Indices

Lamia A. Alamri^1^; Jason Gien^1^; Payton Moody^1^; Blair W. Weikel^2^; Mariana Meyers^1^; Michael V. Zaretsky^1^; Sarkis C. Derderian^1^; Henry L. Galan^3^

*^1^University of Colorado, School of Medicine, Aurora, CO; ^2^University of Colorado, School of Medicine and Colorado Fetal Care Center, Aurora, CO; ^3^University of Colorado, School of Medicine, Children's Hospital of Colorado, Aurora, CO*

8:15 AM ‐ 8:30 AM

**Objective**: Prognosis and management of prenatally diagnosed congenital diaphragmatic hernia (CDH) is reliant on ultrasound (US) observed‐to‐expected lung‐to‐head ratio (O/E LHR) and MRI indices (percent predicted lung volume; PPLV). These indices can conflict with one another (e.g. severe by MRI and moderate by US, or vice versa), creating a clinical dilemma. Our objective was to determine whether discordant prognostic indices are more predictive of a moderate or a severe case.

**Study Design**: This retrospective study included all severe and moderate CDH cases managed at a single fetal care center from 2012‐2023. Severe parameters were defined as a MRI PPLV < 15% and U/S O/E LHR value < 25%. Discordant MRI and US indices (DIS) were defined as either one falling into the severe category and the other into the moderate category, and they were grouped together. Concordant Severe (CSev) and Concordant Moderate (CMod) were defined as MRI and US both falling into the severe or moderate category, respectively. The primary outcome was extracorporeal membrane oxygenation (ECMO) use. Categorical and continuous variables were compared using chi‐square or Fisher's exact tests or Wilcoxon rank sum test as appropriate. Differences between groups were assessed with pair‐wise comparisons and a Bonferroni correction (p< 0.017).

**Results**: Of 82 CDH cases, 27 were in the DIS group, 36 in the CMod group and 19 in the CSev group. Primary and secondary outcomes are shown in the Table 1a. ECMO use was similar for DIS (78%) and CSev (95%) groups, and both were significantly higher (Table 1b) than the CMod group (45%). Survival was highest and mechanical ventilation was lowest in the CMod group (Table 1 & Figure 1). Duration on ECMO and hospital length of stay were similar among all groups.

**Conclusion**: Discordant prenatal MRI and US CDH indices can lead to uncertainty and affect optimal patient counseling and management. Our results suggest that MRI and US modalities which show discordant CDH prognostic indices, predict a severe CDH phenotype.

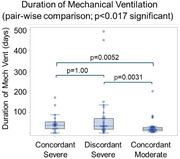

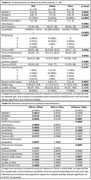

## 88 | Tracking Twin Growth from Start to Finish in 3D

Jessica L. Gleason^1^; Zhen Chen^2^; Rajeshwari Sundaram^2^; Kathryn A. Wagner^3^; Daniel He^4^; Wesley Lee^5^; Roger Newman^6^; Seth Sherman^7^; Edward Chien^8^; Robert Gore‐Langton^7^; Luis F. Goncalves^9^; Katherine L. Grantz^1^

*^1^Epidemiology Branch, NICHD‐NIH, Bethesda, MD; ^2^National Institute of Child Health and Human Development, Bethesda, MD; ^3^Massachusetts College of Pharmacy and Health Sciences, Boston, MA; ^4^The Prospective Group, Fairfax, VA; ^5^Baylor College of Medicine, Houston, TX; ^6^Medical University of South Carolina, Columbia, SC; ^7^The Emmes Company, Rockville, MD; ^8^Cleveland Clinic, Cleveland, OH; ^9^Phoenix Children's Hospital, Phoenix, AZ*

8:30 AM ‐ 8:45 AM

**Objective**: An emerging paradigm attributes 3^rd^ trimester deceleration in fetal growth in uncomplicated twin pregnancies to a normal evolutionary adaptive process rather than pathologic growth restriction. However, long‐term studies tracking child growth are needed since growth restriction in singletons is associated with increased risk for obesity and cardiometabolic dysfunction later in life. Few such studies exist, and none continue through adolescence or have evaluated fetal fat body composition which may provide important insights.

**Study Design**: In the NICHD‐Fetal 3D Study (n = 2919), we compared marginal means of total, fat, and lean fractional thigh volumes between twins and singletons between 15‐37 weeks’ gestation (wks) estimated from linear mixed models. Additionally, in US and UK nationally representative prospective datasets (n = 30,854), we similarly compared growth trajectories of twins and singletons between 0‐18 years (y) and estimated adjusted risk ratios (aRR) of obesity overall and at different stages of development, based on typical ages of puberty for boys and girls.

**Results**: *In utero*, twins had smaller thigh volumes beginning at 15 wks (difference = ‐0.12 cm^3^; 95% CI ‐0.16, ‐0.08) through 37 wks (difference = ‐7.8 cm^3^; CI ‐11.9, ‐3.8), with 2.7‐4.2% less thigh fat (15‐37 wks) and less lean tissue by 0.18‐1.85 cm^3^ (23‐37 wks) than singletons (Figure 1). From birth through age 18y, twins never catch up to singletons, remaining consistently 1 cm shorter, 1‐2 kg lighter, and a lower BMI (Figure 2). Accordingly, twins’ risk of obesity was 25% lower between age 3‐18y; 27% lower (aRR = 0.73; CI 0.54, 0.99) before puberty, 29% lower (aRR = 0.71; CI 0.51, 0.99) during puberty, and 10% lower (aRR = 0.90; CI 0.55, 1.04) after puberty.

**Conclusion**: Twins had proportionally less fat tissue accumulation *in utero* compared to singletons starting at 15 weeks and were not at an increased risk of childhood obesity. Rather, twins had shorter stature and were thinner through age 18y. Persistent differences in twin size across gestation and through age 18y supports the concept of an early adaptive programming mechanism.

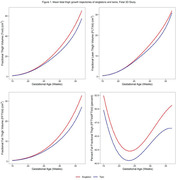

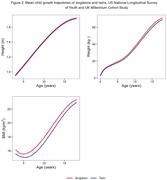

## 89 | Proteomic Analysis of Monochorionic Pregnancies with twin‐to‐twin Transfusion Syndrome and Selective Fetal Growth Restriction

Jessian L. Munoz; Cara Buskmiller; Ahmed A. Nassr; Magdalena Sanz Cortes; Michael A. Belfort; Roopali V. Donepudi

*Texas Children's Hospital and Baylor College of Medicine, Houston, TX*

8:45 AM ‐ 9:00 AM

**Objective**: One third of monochorionic twin pregnancies are complicated by twin‐to‐twin transfusion syndrome (TTTS) and/or selective fetal growth restriction (sFGR). While standard treatment of TTTS is fetoscopic laser photocoagulation (FLP), concomitant sFGR results in greater rates of fetal demise and adverse pregnancy outcomes. Our objective was to characterize the molecular pathophysiology of TTTS, sFGR and the relationship when both are present.

**Study Design**: This study was a prospective cohort analysis at our Fetal Center from 2022‐2024. Maternal plasma specimens and amniotic fluid samples were collected from patients undergoing FLP for TTTS with and without sFGR. Amniotic fluid total protein was assessed by Bradford assay and quantitative proteomic pathway analysis was performed using the Luminex platform. Maternal plasma proteins were then analyzed by ELISA to assess for possible circulating biomarkers.

**Results**: Amniotic fluid specimens were analyzed from 36 subjects (18 isolated TTTS and 18 TTTS + sFGR). No differences were noted in gestational age of FLP. Proteomic analysis (figure 1) showed significant downregulation of Fibroblast Growth Factor (FGF‐2, p = 0.02), Endoglin (Eng, p = 0.04) and Vascular Endothelial Growth Factor C (VEGF‐C, p = 0.01) in pregnancies complicated by TTTS with sFGR when compared to isolated TTTS. No differences were noted in other vascular or growth factors. Pathway interaction analysis revealed a linear relationship among these proteins in neovascularization and cell growth pathways. Maternal plasma proteomic analysis showed no difference in protein expression among groups.  

**Conclusion**: In this cohort of monochorionic pregnancies complicated by TTTS and TTTS with sFGR, the pro‐vascular and pro‐growth pathway of FGF‐2/Endoglin/VEGF was significantly downregulated, suggesting these may contribute to the sFGR pathology beyond TTTS. These findings were limited to the amniotic cavity and not identified in maternal circulation. Further research on the downstream molecular mechanism of this proteomic pathway may provide additional targets to assess and treat underlying pathology.

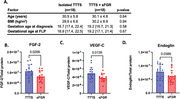

## 90 | Chronotropic Rescue Prior to Cord Clamping in Fetal Complete AV Block and Severe Bradycardia

Samantha Holmes^1^; Emily Bucholz^1^; Camila Londono‐Obregon^1^; Jason Gien^1^; Nicholas Behrendt^1^; Michael V. Zaretsky^1^; Henry L. Galan^2^; Bettina Cuneo^3^

*^1^University of Colorado, School of Medicine, Aurora, CO; ^2^University of Colorado, School of Medicine, Children's Hospital of Colorado, Aurora, CO; ^3^University of Arizona, Tucson, AZ*

9:00 AM ‐ 9:15 AM

**Objective**: Fetal complete AV block (CAVB) occurs in 2% of pregnancies with Anti‐Ro antibodies or inherited arrhythmias. Only about 10% have a ventricular (V) rate of < 50 beats per minute (bpm) but case mortality rate is 42%. We hypothesized that survival of fetal CAVB with V ≤52 bpm prior to delivery would improve with chronotropic rescue using epinephrine (epi) or ventricular pacing at or after cesarean delivery and prior to cord clamping.

**Study Design**: Single center retrospective series of fetal CAVB divided into 2 groups based on V rate at time of delivery. Group 1 consists of V rates > 52bpm receiving standard delivery room (DR) resuscitation at delivery. Group 2 consists of V rates ≤52 bpm receiving either standard resuscitation or chronotropic rescue prior to cord clamping and delivery. Maternal, fetal and neonatal medical records and echocardiograms were reviewed for atrial/ventricular rates, cardiac function and survival or demise. We calculated median for both groups.

**Results**: From 2013‐2024, 19 fetal CAVB subjects (10 in group 1, 9 in group 2), 18 with maternal Anti‐Ro antibodies and one with an inherited arrhythmia, were delivered (Figure 1). Group two delivered and presented at an earlier gestational age (GA), had lower V rates at presentation and received more in utero terbutaline than in group one. All in group one survived after standard DR resuscitation. In group 2a, three died after standard DR resuscitation and pacing within 30 minutes of birth because of pulseless electrical activity: their V rates decreased 50 (39‐51) bpm and cardiac function became severely depressed after cord clamping. In group 2b, 6/6 survived after receiving high dose epinephrine (n = 5) or EXIT to pacing prior to cord clamping (n = 1); their V rate increased 63 (57‐70) bpm and cardiac function was normal after cord clamping (Figure 2). All with chronotropic rescue were successfully paced at < 6 hours.

**Conclusion**: Chronotropic rescue prior to cord clamping may improve survival by increasing V rate and maintaining cardiac function in CAVB with ventricular rates ≤52 bpm and a history of in utero terbutaline treatment.

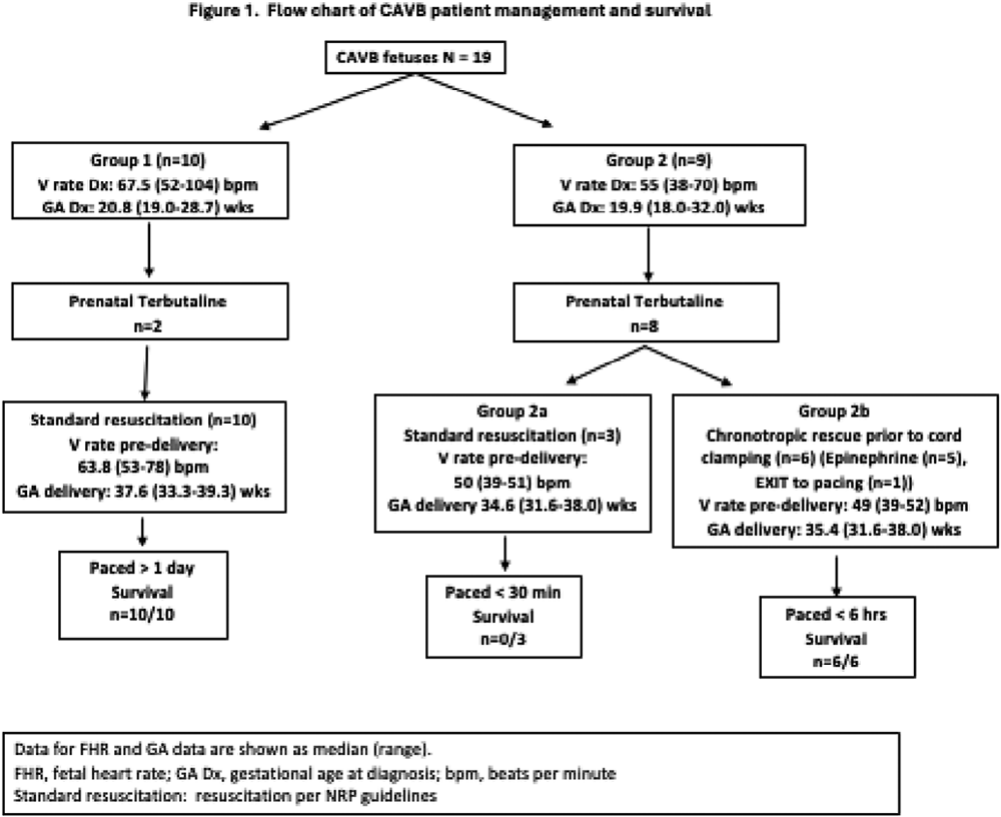

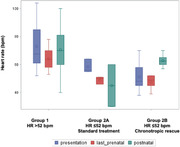

## 91 | Impact of a Synthetic Amniotic Fluid Upon Fetal Lung Development

Stephanie Finoti^1^; Braxton Forde^2^; Samuel Martin^3^; Marc Oria^1^; Jose L. Peiro^4^

*^1^University of Cincinnati, Cincinnati, OH; ^2^University of Cincinnati College of Medicine, Cincinnati, OH; ^3^Cincinnati Children's Hospital Medical Center, Cincinnati, OH; ^4^Cincinnati Children's Fetal Care Center, Cincinnati, OH*

9:15 AM ‐ 9:30 AM

**Objective**: Serial amnioinfusions in the setting of anyhydramnios seek to promote fetal lung development, however these procedures can be fraught with complications, and the fluid infused (Normal Saline‐NS or Lactated Ringer's‐LR) is not designed for the in‐utero environment. We previously designed a synthetic amniotic fluid (Amnio‐well ‐ AW), which minimizes reactive oxygen species damage to the amniotic membrane. Given the interplay between the amniotic fluid and fetal lungs, we sought to evaluate the impact of AW on fetal lung development.

**Study Design**: At E17.5, pregnant rats underwent laparotomy to amniotic fluid replacement with either NS, LR, AW, or AW plus epidermal and fibroblast growth factor (AW++), with sham surgery as a control. Fetal lungs were harvested at E20.5. Histology was evaluated by H&E staining, using morphometric measurement of fractional airspace to estimate growth. Gene expression for surfactant A, B, and C (SP‐A, SP‐B, SP‐C) was compared between groups via reverse transcriptase PCR and immunofluorescence. Inflammatory gene panels were run to identify patterns in abnormal inflammation in the various treatment groups.

**Results**: Fetal lungs from NS and LR were noted to have increased edema, macrophage infiltration, and decreased airspace (p< 0.001) (Figure 1).  When evaluating surfactant expression, there was increased SP‐B, SP‐C expression with AW relative to control, significantly decreased SP‐A, SP‐B, SP‐C expression with both NS and LR, and mildly decreased SP‐A, SP‐B, SP‐C with AW++ (Figure 2). Inflammatory gene profiling revealed marked alterations in histamines, annexins, phospholipase, and immune cell recruitment in NS and LR, indicating abnormal cell membrane integrity (Figure 2). The closest profile to control was AW, with altered gene expression in 10/94 genes (9 upregulated, 1 downregulated), vs 41 genes with NS (22 up, 19 down), 33 with LR (14 up, 19 down), and 12 with AW++ (10 up, 2 down).

**Conclusion**: A synthetic amniotic fluid leads to decreased lung inflammatory profiles and improved surfactant expression compared to commercially available fluids when used for amnioinfusion.

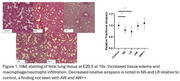

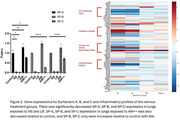

## 92 | Effect of Very Preterm Delivery on Outcomes Following in‐Utero Spina Bifida Repair

Michael V. Zaretsky^1^; Virginia Lijewski^1^; Jagruti Anadkat^2^; Kelly A. Bennett^3^; Yair J. Blumenfeld^4^; Christopher Q. Buchanan^5^; Caitlin M. Clifford^6^; Timothy Crombleholme^7^; Samer Elbabaa^8^; Stephen P. Emery^9^; William H. Goodnight^10^; Hanmin Lee^11^; Joseph B. Lillegard^12^; Foong‐Yen Lim^13^; Francois Luks^14^; Jena L. Miller^15^; Ueli Moehrlen^16^; Julie Moldenhauer^17^; Anita J. Moon‐Grady^11^; Mauro Schenone^18^; Aimen F. Shaaban^19^; KuoJen Tsao^20^; Tim Van Mieghem^21^; Amy J. Wagner^22^; On behalf of the fMMC Consortium sponsored by NAFTNet

*^1^University of Colorado, School of Medicine, Aurora, CO; ^2^Washington University School of Medicine, St. Louis, MO; ^3^Vanderbilt University Medical Center, Nashville, TN; ^4^Children's Hospital at Stanford, Palo Alto, CA; ^5^St. Louis Fetal Care Institute, St. Louis, MO; ^6^University of Michigan Medical Center, Ann Arbor, MI; ^7^Connecticut Children's Fetal Care Center, Hartford, CT; ^8^Orlando Health Arnold Palmer Hospital for Children, Orlando, FL; ^9^University of Pittsburgh, Pittsburgh, PA; ^10^University of North Carolina at Chapel Hill, Chapel Hill, NC; ^11^University of California, San Francisco, San Francisco, CA; ^12^Midwest Fetal Care Center, Minneapolis, MN; ^13^Cincinnati Children's Hospital, Cincinatti, OH; ^14^Fetal Treatment Program of New England, Providence, RI; ^15^Johns Hopkins Medicine, Baltimore, MD; ^16^Children's Hospital Zurich, Zurich; ^17^Children's Hospital of Philadelphia, Philadelphia, PA; ^18^Mayo Clinic, Rochester, MN; ^19^Chicago Institute for Fetal Health, Chicago, IL; ^20^McGovern Medical School at the University of Texas Health Science Center at Houston (UTHealth) University of Texas Health Science Center, Houston, TX; ^21^Mount Sinai Hospital and University of Toronto, Toronto, ON; ^22^Children's Hospital of Wisconsin Fetal Concerns Center, Milwaukee, WI*

9:30 AM ‐ 9:45 AM

**Objective**: In‐utero fetal spina bifida repair decreases the risk for ventriculoperitoneal shunt (VP shunt) placement, reverses hindbrain herniation, and improves motor function with a higher likelihood of independent ambulation. The goal of this project was to assess pregnancy and neonatal outcomes among individuals who delivered very preterm (< 30 weeks of gestation (wk)) from the fMMC Consortium Registry sponsored by the North American Fetal Therapy Network (NAFTNet).

**Study Design**: The NAFTNet fMMC registry includes prospectively collected data from 2011 to 2024. Three cohorts of patients were categorized by gestational age (GA) of delivery and compared: < 30 wk, 30‐34 wk, and > 34 wk.  Comparisons between groups for maternal and delivery outcomes were completed for the entire cohort and then analyzed for those with available shunt data. Chi‐square or Fisher's exact tests were used to compare categorical variables, and Kruskal‐Wallis tests were used to compare continuous variables. p‐values < 0.05 were considered significant.

**Results**: A total of 1,213 patients were available for analysis (< 30 wk n = 111, 30‐34 wk n = 323, >34 wk n = 779).  Overall mortality was 2.2%, 85% of which occurred in the < 30 wk cohort (Table 1) in which perinatal mortality was 21%. Latency from fetal surgery to delivery averaged 2.6 wks for the < 30 wk group. Rates of abruption, chorioamnionitis, and spontaneous labor were higher compared to later GA cohorts. Neonatal morbidity was also significantly higher for sepsis, apnea, respiratory distress, patent ductus arteriosus, and periventricular leukomalacia in the < 30 wk cohort. VP shunt data was available for 677 patients (< 30 wk n = 53, 30‐34 wk n = 186, >34 wk n = 438). A similar rate of VP shunt placement was seen between GA groups (Table 2). 

**Conclusion**: Very preterm birth following in‐utero spina bifida repair results in significant perinatal mortality and neonatal morbidity.  Despite very preterm birth, the reduction in VP shunt rates, a benefit of in utero repair, appears to be preserved.  Further data is required to analyze the impact on ambulation and developmental outcomes.

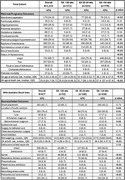

## 93 | In‐Utero Ventriculosubgaleal Shunt Placement ‐ a Fetal Lamb Model of Induced Hydrocephalus

Shohra Qaderi^1^; Weston Northam^1^; Soner Duru^2^; Eyal Krispin^3^; Syril James^4^; Jose L. Peiro^5^; Braxton Forde^6^; Hamidreza Forourtan^7^; Ramen H. Chmait^8^; Scott A. Shainker^9^; Cassandra R. Duffy^9^; Nikan Zargarzadeh^1^; Ali Javinani^1^; Arthur Nedder^10^; Brittany Pattison^10^; lana Vasung^10^; Ryne A. Didier^10^; Michaela K. Farber^11^; Sebastian Seifert^12^; Darren B. Orbach^10^; P. Ellen Grant^10^; Benjamin C. Warf^10^; Yves Ville^13^; Alireza A. Shamshirsaz^14^

*^1^Boston Children's Hospital, Harvard Medical School, Boston, MA; ^2^Cincinnati Children's Hospital, Cincinnati, OH; ^3^Harvard Medical School, Boston, MA; ^4^Hôpital Necker‐Enfants Malades, Paris, Ile‐de‐France; ^5^Cincinnati Children's Fetal Care Center, Cincinnati, OH; ^6^University of Cincinnati College of Medicine, Cincinnati, OH; ^7^Laparoscopy research center, Shiraz, Fars; ^8^Keck School of Medicine, University of Southern California, Los Angeles, CA; ^9^Beth Israel Deaconess Medical Center, Boston, MA; ^10^BCH, Boston, MA; ^11^BWH, Boston, MA; ^12^Brigham & Women's Hospital, Boston, MA; ^13^University and Necker‐Enfants Malades Hospital, Paris, Ile‐de‐France; ^14^Boston Children's Hospital, Brigham and Women's Hospital, Beth Israel Deaconess Medical Center, Harvard Medical School, Boston, MA*

9:45 AM ‐ 10:00 AM

**Objective**: Irreversible brain damage due to congenital hydrocephalus may be improved with prenatal intervention. While ventriculosubgaleal shunting (VSGS) is commonly performed postnatally in premature infants, in‐utero placement is unexplored. For the first time, we evaluate the feasibility of VSGS in‐utero using a lamb model.

**Study Design**: Hydrocephalus was induced in 14 fetal lambs by injecting 1.5 mL of BioGlueⓇ into the cisterna magna (Fig 1a‐1d) between days 80‐90 of gestation. Ten to 16 days later, ultrasound was performed to confirm hydrocephalus. Afterward, VSGS was placed by creating a subgaleal pocket (Fig 1e), inserting a standard ventricular catheter (Fig 1f), tunnelling under the scalp (Fig 1g), and securing with a silk suture (Fig 1h), with the other end in a lateral ventricle (Fig 1i). Near term, the ewes were sacrificed. Hysterotomy was performed to extract the lambs. Lambs were evaluated through gross inspection, and postmortem MRI was performed. Neuropathology is pending.

**Results**: Ten ewes with 14 fetuses underwent hydrocephalus induction (median 86 days) by either hysterotomy (2/14) or transuterine (12/14) methods. Two fetuses were macerated, but hydrocephalus was confirmed in 11/12 surviving fetuses (3 severe, 4 moderates, 4 mild). A VSGS was successfully placed in all 12 (median 104 days) (Fig 1e‐1h). One with severe hydrocephalus died after the procedure, 3 were aborted and 1 was macerated at hysterotomy. All 7/14 lambs that survived to near term showed scalp healing (Fig 1l). All 7 VSGS remained under the scalp without migration (Fig 1j‐1k) (Table 1). Postmortem MRI showed improved hydrocephalus in 4/7 cases (Table 1).

**Conclusion**: Prenatal placement of VSGS proved feasible in this animal model. The procedure resulted in a 50% fetal loss, which may be due to procedural factors or stress from repeated procedures. Improved hydrocephalus occurred in 57% of survivors. To improve success, future studies will investigate the efficacy and safety of VSGS using less invasive approaches, including fetoscopic placement of VSGS.

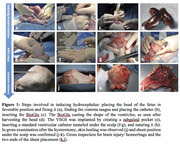

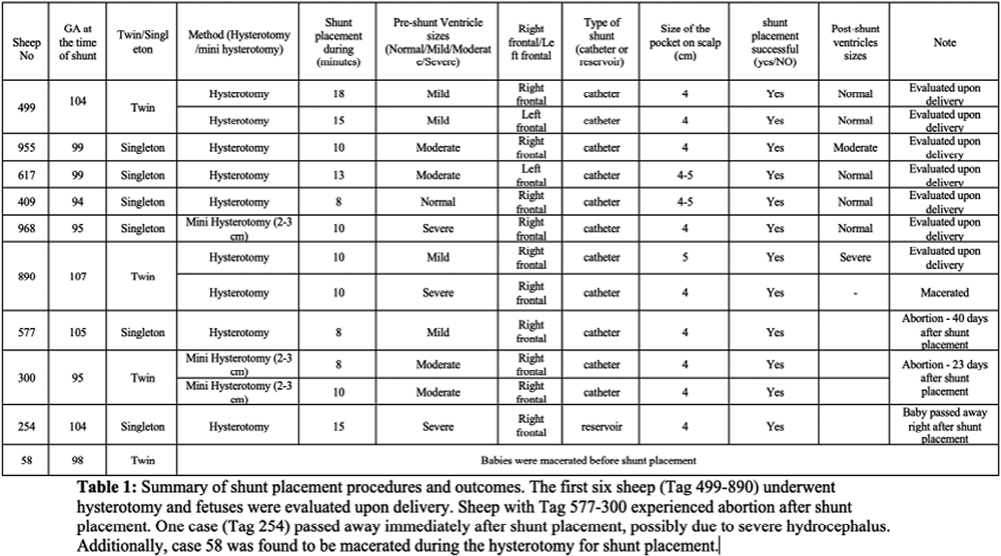

## 94 | Role of Placental Superficial Anastomoses in Twin‐Twin Transfusion Syndrome (TTTS)

Ramen H. Chmait^1^; Lisa M. Korst^2^; Arlyn Llanes^1^; Kristine R. Rallo^1^; Andrew H. Chon^3^; Martha A. Monson^4^; Ruben A. Quintero^5^

*^1^Keck School of Medicine, University of Southern California, Los Angeles, CA; ^2^Childbirth Research Associates, North Hollywood, CA; ^3^Oregon Health & Science University, Portland, OR; ^4^Intermountain Healthcare, University of Utah Health, Salt Lake City, UT; ^5^The Fetal Institute, USFETUS Research Consortium, Miami, FL*

10:00 AM ‐ 10:15 AM

**Objective**: To test whether the type and number of superficial anastomoses [arterio‐arterial (AA) and veno‐venous (VV)] were associated with twin survival.

**Study Design**: This is a post hoc analysis of data collected in the Sequential Trial, a randomized controlled trial (RCT) comparing the sequential and selective laser techniques for TTTS patients at 16‐26 gestational weeks. Patients received either the original (selective) or a modified selective (sequential) laser surgery, in which arteriovenous (AV) anastomoses with unidirectional blood flow from donor to recipient are laser‐ablated first, followed by ablation of AV anastomoses from recipient to donor. This theoretically allows for a net intraoperative blood transfer from the hypervolemic recipient to the hypovolemic donor twin. Those with only 1 AA (AA‐1) vs 2 or more AA (AA‐2) were identified, and we tested for association for twin survival at birth.

**Results**: Of 642 patients in the RCT, 466 (72.6%) had AV anastomoses only (donor survival 90.3%) and 176 (27.4%) had both AV and superficial anastomoses (donor survival 70.5%) (*P*< .0001). Of the 176 with superficial anastomoses, 112 (63.6%) had AA only, 40 (22.7%) had both AA and VV, and 24 (13.6%) had VV only. For the 152 patients who had at least 1 AA, donor survival differed based on type and number of superficial anastomoses (Figure). In logistic regression models for donor survival controlling for surgical technique, donor critical abnormal Dopplers, and donor middle cerebral artery peak systolic velocity multiples of the median >1.5, patients with AA‐2 vs AA‐1 were less likely to have donor survival (OR 0.26 [0.08‐0.81], *P* = .0206). Specifically, patients with 2 or more AA and no VV were at risk (Table). The presence of fetal growth restriction was non‐contributory. No differences in recipient survival were noted.

**Conclusion**: The type and number of superficial anastomoses was associated with donor but not recipient twin survival after laser surgery for TTTS. Poor donor twin survival was associated with presence of AA, particularly in cases lacking a corresponding VV.